# The Hebrew Influence

**Published:** 1896-07-18

**Authors:** 


### MODERN SOCIOLOGY.

### THE HEBREW INFLUENCE.

THE greatest of German historians has said that the noblest developments of human nature have resulted from the implanting Semitic seed upon Aryan ground. It is this which gives the history of the Hebrews its peculiar significance and interest in studying the growth of Sociology from its earliest beginnings. Another great German writer, Lessing, in a book published in the eighteenth century, entitled, "The Education of the Human Race," describes the part that the Hebrew nation was intended to play in the general destinies of humanity—that is, that the special mission was the moral and religious elevation of the race. It is this fact that, apart from all theological controversy and discussion, renders the history of the Hebrew race one of abiding interest and importance for all other races of mankind. Our own great historian Gibbon, whose work has been described as the grand bridge over which all future students and readers of history will pass from the ancient into the modern world, in one of the earliest chapters of his great work says Judæa and Phœnicia will never be forgotten, for from the one the world received its religion, from the other its letters, which have been the foundation of its literature. Another most significant fact is, that the Jews alone of all the most ancient nations survive to this day, the same people, with the same religion and the same race, as those who emigrated from Egypt thousands of years ago to escape from the tyranny of the Pharaohs. In one of the very earliest chapters of the Bible it is stated that Noah, when prophesying the future destiny of his sons, said, "God shall enlarge Japhet, and he shall dwell in the tents of Shem." The usual interpretation of this text is understood to be that Japhet shall dwell in the tents of Shem; but the old Hebrew version is, that it is intended to convey the meaning that God Himself shall dwell in the tents of Shem. We quote this familiar text, as it seems to foreshadow in prophecy the great gulf and different characteristics which, up to the present day, have separated the Jewish and the Gentile world. Later on it is said that Abraham was told that "in his seed should all the nations of the earth be blessed." These old texts are worthy of the closest study, as they still exercise a most potent influence over all the modern Mohammedan world, and in spite of the so-called "higher criticism" over a very great part of the modern Christian world also. When we come to the story of the Exodus, and the promulgation of the Mosaic law, we feel somewhat more in touch with the modern civilisation over which it has exerted so great an influence. Its sanitary enactments have been acknowledged to be thoroughly sound by the greatest of modern authorities, and it contains the germs of progress from which it may be said that many of the most important changes in modern legislation have arisen. To take only one instance as an example. In Leviticus xxv., when the ceremonies of the Jubilee year are described, it is ordained that every man shall be returned unto his possessions and unto his family. This is the most ancient of all records which aims a blow at slavery, which "peculiar" institution, as it has been called, is, perhaps, of all others the one which most sharply divides the social conceptions of the ancient and the modern world. There were innumerable bodies of Jews scattered throughout the Roman and even the Parthian Empire. At the period just before the introduction of Christianity, the Jews played a peculiar part among the nations. Without a strong political organisation of their own, and yet one of the most firmly welded together races of the world, they helped to prepare the way for that cosmopolitanism which for some centuries made the Roman world a term almost synonymous with civilized humanity.

During the Middle Ages the Jews had little or no influence upon the social development of Europe, unless, indeed, their commercial power, and their scientific, and especially medical knowledge, may be supposed to have exerted some indirect influence. But in the present century they have strongly influenced European opinion on these subjects.

It is a curious and interesting fact that their social freedom in England began during the rule of Cromwell, and in France at the period of the Revolution. In Germany they may claim to be the founders of the Socialist party, as it was chiefly through the influence of Karl Marx and of Lassalle that this political party, now the strongest of its kind in Europe, was founded. But Socialism is only a form, and probably only a passing phase of sociology, standing on the same level in this respect with Republicanism and Monarchy. The curious position of the Jews consists in their being a thoroughly Oriental race, and yet one in constant contact with every form of Western civilisation in every corner of the world. Their democratic policy of the ancient days may account for this to some extent, as every son of Israel was equal before the Almighty, as we can easily gather from numerous texts in the Old Testament. When this equality had to be extended to all mankind, without distinction of race, then the separation of the Jew and the Gentile became more accentuated and acute than ever. But the Jewish race, through their peculiar combination of qualities, may yet be destined to play a great part in the evolution of sociological ideas in the future. They represent the one Eastern nation that is in constant and immediate touch with every part of the West. While they have preserved their own nationality in a way, and under such difficulties, as have affected no other nation in history, they have still to a very great extent become mixed up with the social life of every other nation with any pretence to civilisation upon the face of the earth. And when to this fact is added the fact that they have come down from the earliest antiquity as an unmixed race, if any credence is to be given to the theory of heredity, we cannot but believe that their influence will be felt in every future development of sociology to a very marked degree. The question of the influence of the doctrines of Christianity upon the development of sociology, as they are expounded in the New Testament, will be dealt with in a future article, as this influence is in many respects, although starting from the same origin, profoundly antagonistic to the Jewish influence.

